# Retraction Note: Circular RNA circStag1 promotes bone regeneration by interacting with HuR

**DOI:** 10.1038/s41413-024-00348-2

**Published:** 2024-06-24

**Authors:** Gaoyang Chen, Canling Long, Shang Wang, Zhenmin Wang, Xin Chen, Wanze Tang, Xiaoqin He, Zhiteng Bao, Baoyu Tan, Jin Zhao, Yongheng Xie, Zhizhong Li, Dazhi Yang, Guozhi Xiao, Songlin Peng

**Affiliations:** 1 Department of Spine Surgery and Institute for Orthopaedic Research, the Second Clinical Medical College of Jinan University (Shenzhen People’s Hospital), Shenzhen Key Laboratory of Musculoskeletal Tissue Reconstruction and Function Restoration, Shenzhen, 518020 China; 2 The First Affiliated Hospital, Southern University of Science and Technology, Shenzhen, 518055 China; 3 The First Affiliated Hospital, Jinan University, Guangzhou, 510630 China; 4 School of Medicine, Southern University of Science and Technology, Guangdong Provincial Key Laboratory of Cell Microenvironment and Disease Research, Shenzhen Key Laboratory of Cell Microenvironment, Shenzhen, 518055 China

**Keywords:** Osteoporosis, Bone

Retraction Note to: *Bone Research* 10.1038/s41413-022-00208-x, published online 31 March 2022

The authors have retracted this article. After publication, concerns were raised regarding a number of overlapping images in this article, as well as the reuse of data from the authors’ earlier article.^1^ The authors have checked the underlying data and determined that there was inadvertent mixing of images during the preparation of the submitted materials due to the extensive volume of images involved in the study.

The authors are reviewing the data and intend to submit a revised manuscript for peer review in due course.

All authors agree to this retraction.
